# CRISPR-Cas System: An Approach With Potentials for COVID-19 Diagnosis and Therapeutics

**DOI:** 10.3389/fcimb.2020.576875

**Published:** 2020-11-02

**Authors:** Prashant Kumar, Yashpal Singh Malik, Balasubramanian Ganesh, Somnath Rahangdale, Sharad Saurabh, Senthilkumar Natesan, Ashish Srivastava, Khan Sharun, Mohd. Iqbal Yatoo, Ruchi Tiwari, Raj Kumar Singh, Kuldeep Dhama

**Affiliations:** ^1^ Amity Institute of Virology and Immunology, Amity University, Noida, India; ^2^ Division of Biological Standardization, Indian Council of Agricultural Research-Indian Veterinary Research Institute, Bareilly, India; ^3^ College of Animal Biotechnology, Guru Angad Dev Veterinary and Animal Science University, Ludhiana, India; ^4^ Laboratory Division, Indian Council of Medical Research—National Institute of Epidemiology, Ministry of Health & Family Welfare, Chennai, India; ^5^ Academy of Scientific and Innovative Research (AcSIR), CSIR-HRDC Campus, Ghaziabad, India; ^6^ Plant Molecular Biology and Biotechnology Division, CSIR-National Botanical Research Institute, Lucknow, India; ^7^ Indian Institute of Public Health Gandhinagar, Gandhinagar, India; ^8^ Division of Surgery, ICAR-Indian Veterinary Research Institute, Bareilly, India; ^9^ Division of Veterinary Clinical Complex, Faculty of Veterinary Sciences and Animal Husbandry, Sher-E-Kashmir University of Agricultural Sciences and Technology of Kashmir, Srinagar, India; ^10^ Department of Veterinary Microbiology and Immunology, College of Veterinary Sciences, UP Pandit Deen Dayal Upadhayay Pashu Chikitsa Vigyan Vishwavidyalay Evum Go-Anusandhan Sansthan (DUVASU), Mathura, India; ^11^ Division of Veterinary Biotechnology, ICAR-Indian Veterinary Research Institute, Bareilly, India; ^12^ Division of Pathology, ICAR-Indian Veterinary Research Institute, Bareilly, India

**Keywords:** coronavirus, pandemic (COVID-19), SARS-CoV-2, CRISPR, diagnosis, therapeutic

## Abstract

COVID-19, the human coronavirus disease caused by SARS-CoV-2, was reported for the first time in Wuhan, China in late 2019. COVID-19 has no preventive vaccine or proven standard pharmacological treatment, and consequently, the outbreak swiftly became a pandemic affecting more than 215 countries around the world. For the diagnosis of COVID-19, the only reliable diagnostics is a qPCR assay. Among other diagnostic tools, the CRISPR-Cas system is being investigated for rapid and specific diagnosis of COVID-19. The CRISPR-Cas-based methods diagnose the SARS-CoV-2 infections within an hour. Apart from its diagnostic ability, CRISPR-Cas system is also being assessed for antiviral therapy development; however, till date, no CRISPR-based therapy has been approved for human use. The Prophylactic Antiviral CRISPR in huMAN cells (PAC-MAN), which is Cas 13 based strategy, has been developed against coronavirus. Although this strategy has the potential to be developed as a therapeutic modality, it may face significant challenges for approval in human clinical trials. This review is focused on describing potential use and challenges of CRISPR-Cas based approaches for the development of rapid and accurate diagnostic technique and/or a possible therapeutic alternative for combating COVID-19. The assessment of potential risks associated with use of CRISPR will be important for future clinical advancements.

## Introduction

Coronaviruses (CoVs), derived from the Latin word *corona* (crown), are a group of viruses that have crown-like spikes on their outer surface (Rabi et al., 2020). CoVs spread extensively among humans, birds, and other mammals and cause respiratory, neurologic, enteric, and hepatic diseases (Weiss and Leibowitz, 2011). The emergence of a novel human CoV, which had not been previously isolated from human or any other animal species, was reported in Wuhan, China on December 8, 2019. The disease caused by the novel CoV was named as “COVID-19” by World Health Organization (WHO). The virus causing COVID-19 resembles severe acute respiratory syndrome coronavirus (SARS-CoV), therefore, it was termed SARS-CoV-2 by the International Committee on Taxonomy of Viruses (ICTV). COVID-19 was declared to be a Public Health Emergency of International Concern (PHEIC) on January 30, 2020, and pandemic on March 11, 2020 by the WHO due to its rapid spread around the globe within a concise period.

As of now, over 24.9 million confirmed cases and approximately 0.84 million deaths have been reported from over 215 countries across the world (WHO). Several speculations have been made about the origin of the SARS-CoV-2 virus. Two notable genetic features of the SARS-CoV-2 virus has been reported; first is the mutation in receptor-binding domain (RBD) of the spike protein and second is the introduction of polybasic cleavage site (RRAR) at the junction of S1 and S2 which are the two subunits of spike protein (Malik et al., 2020; Rabaan et al., 2020). Similarly, population based analyses of beta-coronaviruses using RNA-dependent RNA-polymerase (RdRp) showed that SARS-CoV-2 represents a homogeneous population which have evolved from RaTG13, the bat coronavirus, which is ancestrally related to Pangolin-CoVs (Malik et al., 2020). Likewise, studies on the molecular mechanisms of SARS-CoV-2 and SARS-CoV infection in human beings Molecular Dynamics (MD) report that the binding affinity of SARS-CoV-2 to the ACE2 receptor is higher than SARS-CoV, a fact which can be attributed to the enhanced infectious nature of SARS-CoV-2 (Rabaan et al., 2020).

Seeing the emergence of SARS-CoV-2, an understanding of the zoonotic transfer of viruses may help in preventing such events in future. Three major factors including behavioral changes in human beings, environmental change and change in diversity of microorganisms can be attributed to the emergence/re-emergence of infectious diseases. Such changes are considered to have a synergistic effect in increasing the risks for the emergence of pathogens and its transmission to the susceptible hosts, apart from creating a favorable environment for the pathogen to sustain itself in a reservoir host and/or an intermediate host before reaching humans and causing a major devastating effect (Tiwari et al., 2020).

SARS-CoV-2 is a positive sense single-stranded RNA (ssRNA) virus that is highly contagious among human beings. The virus belongs to the *Betacoronavirus* genus of the *Coronaviridae* family (Gorbalenya et al., 2020). Other coronaviruses, viz. MERS-CoV and SARS-CoV, which have had significant impacts with regard to human health, also belong to the same genus. The SARS-CoV-2 genome is approximately 29.9 kilobases in length (Yadav et al., 2020) and encodes four structural proteins, viz. spike protein (S), envelope protein (E), membrane protein (M), and Nucleoprotein (N), apart from sixteen non-structural proteins, including RNA-Dependent RNA Polymerase (Rabaan et al., 2020). Its genome is unique in encoding a polybasic cleavage site in the spike protein, which contributes to the viral pathogenicity and transmissibility (Andersen et al., 2020; Coutard et al., 2020; Jin et al., 2020; Walls et al., 2020). The S, E, and M constitute the surface protein that protrude out on the viral envelope and assist in causing infection in susceptible host cells (Letko et al., 2020).

The transmission of SARS-CoV-2 to susceptible hosts occurs primarily through aerosols originating from coughing and sneezing by an infected individual; however, indirect contact through contaminated surfaces has also been shown to be responsible for its transmission (Morawska and Cao, 2020). The incubation period of the virus in infected hosts ranges from 2 days to 14 days; it causes symptoms such as fever, sore throat, and shortness of breath, but most of the infected individuals remain asymptomatic and act as major sources for virus transmission in communities (Chan et al., 2020; Dhama et al., 2020a; Guan et al., 2020). Reports are there to show that virus may be transmitted from human beings to animals (Leroy et al., 2020) and it has also been suggested that the virus has been transmitted to humans from bats, which represent the natural reservoir of this virus (Dhama et al., 2020b; Lu et al., 2020; Tiwari et al., 2020). Phylogenetic analysis points towards a high resemblance of SARS-like bat coronavirus with SARS-CoV-2, and it is suggested that an unknown intermediate host may have played a role in transmission of the virus to human beings (Malik et al., 2020; Zhou et al., 2020).

SARS-CoV-2 causes flu-like symptoms, and hence, a specific diagnosis of the infection is very important to reduce the mortality rate. Currently, SARS-CoV-2 is detected using real time RT-PCR which is validated by the WHO. However, being an RNA virus, it mutates at a significant rate and may render the present diagnostic tools inefficient in due course of time. A recent study using submitted sequences of SARS-CoV-2 has revealed the mutations in viral genome that have occurred independently multiple times, and almost 80% of such recurrent mutations produce non-synonymous changes that enable the evolution and adaptation of the virus (Dorp et al., 2020).

Accurate diagnosis is very important for the management of COVID-19 as no standard therapy or vaccines are currently available for the disease; currently, only supportive and/or management treatment procedures are being followed worldwide. Therefore, research and development are on fast-track mode for the development of appropriate diagnostics, vaccines, and/or suitable therapeutics (Dhama et al., 2020c; Nguyen et al., 2020).

The devastating effect of SARS-CoV-2 across the world invites a better management strategy that may include both diagnostic as well as therapeutic tools. The use of CRISPR (clustered regularly interspaced short palindromic repeats)-Cas technology could be an effective, novel, and holistic approach to target viral RNA for its degradation and thus, limit the replication of the virus in host cells, resulting in control of its transmission. CRISPR-Cas system has been identified as a part of adaptive immune system in archaea and bacteria against the virus infections (Brouns et al., 2008) and its applications in humans were found later (Jinek et al., 2012). This system is a promising tool to develop novel diagnostics and also for development of therapeutics to eliminate viral infections (Bayat et al., 2018; Jia et al., 2020). A novel antiviral therapy for treating HIV patients has been developed using the CRISPR-Cas technology (Huang et al., 2017; Herrera-Carrillo et al., 2020). Although it did not cure the patient in the first attempt, the therapy was found to be safe as it did not cause any adverse events during the 19 months of follow up (Xu et al., 2019).

In addition, the CRISPR-Cas system is also used for the treatment of cancer and blindness (Lin et al., 2019; Akram et al., 2020). The technology is also being used to develop an efficient diagnostic tool for detecting SARS-CoV-2 in clinical samples. CRISPR technology has been used for the detection of RNA viruses like lymphocytic choriomeningitis virus, influenza A virus and vesicular stomatitis virus, disease diagnosis, and identification of various bacterial pathogens (Chertow, 2018; Freije et al., 2019; Quan et al., 2019). This review article is focuses on the effects and futuristic applications of CRISPR-Cas biology in the development of a potentially safe, secure, and potent antiviral therapy as well as accurate, specific, and sensitive diagnostics for COVID-19, which represent the world’s need of the hour to save the lives of millions who are at risk of acquiring the infection. However, along with its potential applications the clinical applications needs to be ascertained following regulatory approvals.

## Biology of the CRISPR-Cas System

CRISPR-Cas technology is a highly flexible RNA-guided endonuclease (RGEN)-based nucleic acid editing tool that has transformed the field of genomics, gene editing, gene therapy, and genome imaging. The broad range application of this technology provides immense scope to understand and manipulate genetic or epigenetic elements. CRISPR and Cas proteins are the part of natural adaptive immune systems against invading viruses in archae and bacteria (Brouns et al., 2008). CRISPR-Cas loci on the bacterial genome consist of a CRISPR array, which is up to several hundred direct, often palindromic, repeats (35–45 bases) separated by unique spacer sequences (30–40 bases) (Brouns et al., 2008). Adjacent to the CRISPR array, one or more operons having a cluster of Cas genes encoding the effector enzymes of this system are present (Makarova et al., 2013; Makarova et al., 2015). The immune response provided by the CRISPR-Cas system includes three stages, viz. adaptation, pre-CRISPR RNA (crRNA) expression/processing, and interference.

**Figure 1:**
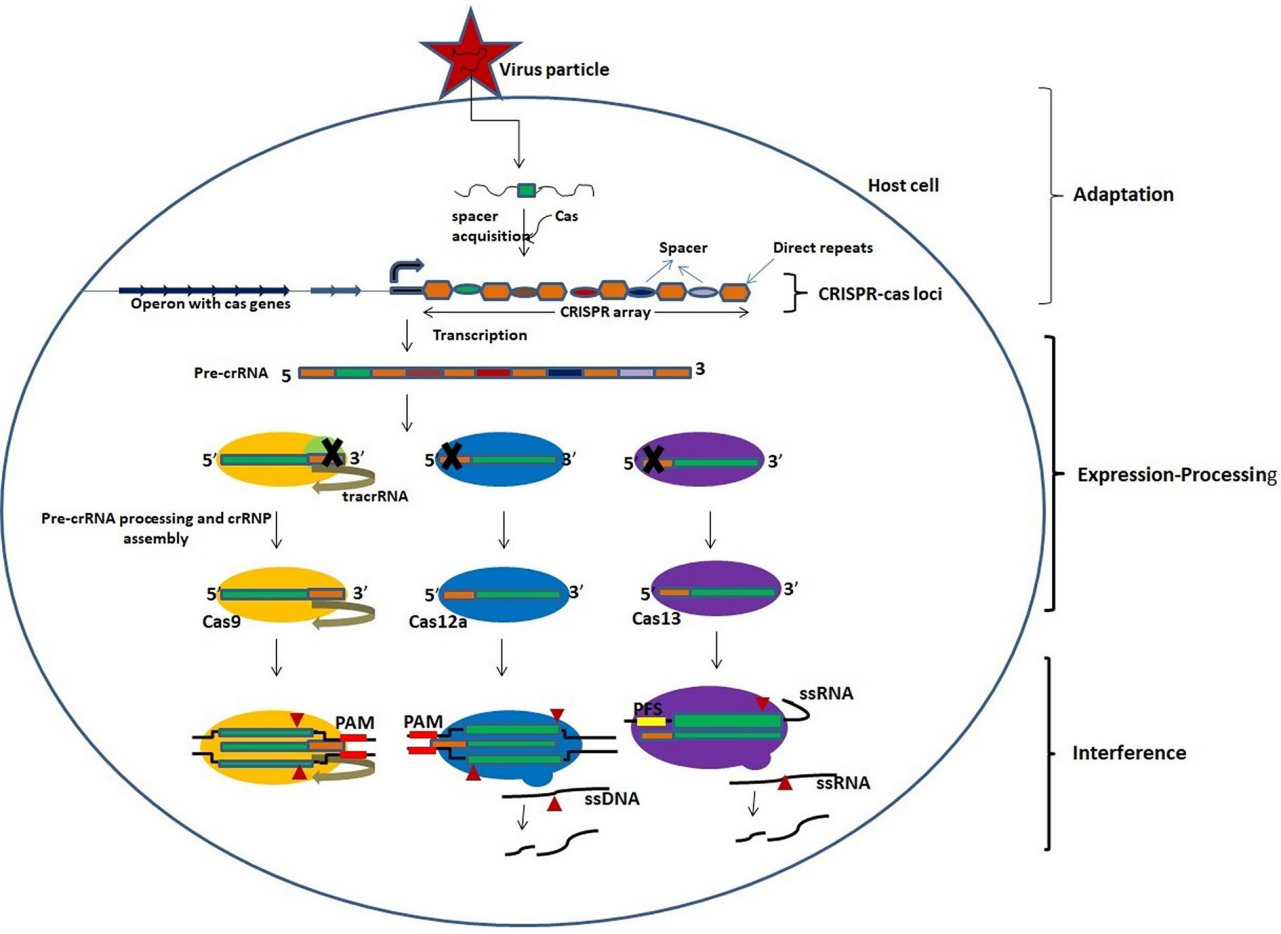
An overview of the activity of CRISPR-Cas system. The activity of CRISPR-Cas system involves three stages: adaptation, expression-processing, and interference. Adaptation: Nucleic acids of invading pathogens are fragmented into short fragments and inserted between two repeats of CRISPR array as a new spacer by the Cas proteins. Expression/processing: Transcription of CRISPR array in the CRISPR locus occurs to produce pre-crRNA which is processed into mature crRNAs by the Cas nucleases. The Cas9 nuclease binds and stabilizes the *tracrRNA: crRNA duplex and processes pre-crRNA by recruiting RNAse III. Cas12a and Cas13 nucleases process the pre-crRNA by themselves. Interference: Mature crRNAs guide the cleavage of target nucleic acids by CRISPR-Cas effector complexes. The cleavage is based on complementarity between the target sequence and the crRNA. *Trans-activating CRISPR RNA that base pairs with the crRNA to form a functional guide RNA (gRNA).

The adaptation stage begins with the expression of a complex of Cas proteins by the CRISPR-Cas loci and binding of these Cas proteins to the target DNA sequence, followed by two double-strand breaks in the target DNA based on the Protospacer Adjacent Motif (PAM) which is a distinct short motif of 2-4 bases. The released segment of the target DNA, called as protospacer, is inserted between two repeats of the CRISPR array and then acts as a new spacer (Amitai and Sorek, 2016; Amitai and Sorek, 2017; Jackson S. A. et al., 2017; Jackson R. N. et al., 2017). The transcription of CRISPR array occurs in expression processing stage to produce a single long pre-crRNA which is processed by a distinct set of Cas proteins to generate mature crRNA (Charpentier et al., 2015; Hochstrasser and Doudna, 2015). This is followed by the interference stage wherein the mature crRNA, bound to the processing complex, acts as guide RNA to recognize similar sequences in the invading viral RNA that is then cleaved and inactivated by one of the Cas proteins (**Figure 1**; **Table 1**) (Plagens et al., 2015; Nishiyama et al., 2017; Nishiyama, 2019).

**Table 1:** Cas proteins and their role.

S.No	Name of protein	Distribution	Role	Function of Cas enzyme	References
1	Cas1	Universal	Spacer Acquisition	Non-specific binding to DNA and RNA; DNase	Wiedenheft et al. (2009) Han et al. (2009)
2	Cas2	Universal	Spacer Acquisition	Specific binding to U rich regions	Beloglazova et al. (2008)
3	Cas3	Type I signature	Interference of target nucleic acid	Act as endonuclease and DNA helicase	Sinkunas et al. (2011)
4	Cas4	Type I, II	Spacer Acquisition	Rec-B like nuclease/exonuclease activity	Zhang et al. (2012)
5	Cas5	Type I	crRNA expression	Endoribonulcease	Koo et al. (2013)
6	Cas6	Type I, III	crRNA expression	Endoribonulcease	Haurwitz et al. (2010)
7	Cas7	Type I	crRNA expression	Endoribonulcease	Staals et al. (2013)
8	Cas8	Type I	crRNA expression	Ruv-C like nuclease; has McrA/HNH nuclease domain	Cass et al. (2015)
9	Cas9	Type II signature	Interference of target nucleic acid	Ruv-C like nuclease; has McrA/HNH nuclease domain; act on DNA	Garneau et al. (2010) Jinek et al. (2012)
10	Cas10	Type III signature	crRNA expression & Interference of target nucleic acid	Endonuclease/has HD nuclease domain	Wang et al. (2019)
11	Cas12	Type V signature	crRNA expression & Interference of target nucleic acid	Endonuclease; act on ssDNA and dsDNA near TTTN; non-specifically cuts ssDNA;	Yan F. et al. (2019)
12	Cas13	Type VI signature	crRNA expression & Interference of target nucleic acid	Endonuclease; act on ssRNA; non-specifically cuts ssRNA	Yan F. et al. (2019)

There are two classes of CRISPR-Cas systems viz. class I and class II. Each of the two classes is further classified into three different subtypes, i.e., Type I, III, and IV in class I and type II, V, and VI in class II (Makarova et al., 2015). The ribonucleoprotein (RNP) complex in the class I system contains multiple protein subunits along with crRNA, while that in the class II system contains only one protein and crRNA to target the invading viral RNAs (Shmakov et al., 2015). The Cas9 protein in type II CRISPR-Cas system processes the pre-crRNA with the help of tracrRNA and RNase III, while the Cas12 and Cas13 proteins of type V and VI systems, respectively, process the pre-crRNA themselves (Deltcheva et al., 2011; Dong et al., 2016; Dong et al., 2017). Cas12a and Cas12b protein cleave the double-stranded target DNA based on recognition by mature crRNA, while the Cas13 protein cleaves the target ssRNA. Cas13 does not require any adjacent protospacer motif (PAM) in the target RNA, while Cas12 requires a PAM in the dsDNA target, but not in ssDNA. A comparison between the nuclease characteristics of Cas12 and Cas13 is provided in **Table 2**.

**Table 2:** Characteristics of Cas12a and Cas13 nucleases (Yan F. et al., 2019).

Nuclease characteristics	Cas12a	Cas13
PAM required	Yes	No
PAM identity	TTTV in dsDNA	Not applicable
Cleavage	Single Staggered cut	Multiple cleavage sites
Target type	ssDNA, dsDNA	ssRNA
Collateral activity	Yes	Yes

The binding of complementary crRNA to the target RNA activates the Cas13 protein, which then degrades the collateral ssRNA (Shmakov et al., 2015; Gao et al., 2016). This property of Cas13 has been demonstrated for the diagnosis of RNA virus infections (Kellner et al., 2019; Kellner et al., 2020). Similar activity has been observed for Cas12 proteins, which can be used for the detection of ssDNA viruses (Li et al., 2018) (**Figure 1**). The Cas13 family contains two HEPN domains that confer RNase activity and exist in 4 subtypes, viz. Cas13a, Cas13b, Cas13c, and Cas13d (O’Connell, 2019). Among all subtypes, Cas13d is important due to its efficient and robust knockdown efficiency and easy viral delivery due to the small coding sequence of its effector domain.

## CRISPR-Cas Applications

The gene-editing application of the CRISPR-Cas system was recognized initially and exploited for developing anti-viral therapies. Later, its application as a gene-detection system was recognized and it has brought about a revolution in diagnostics. Point-of-care CRISPR diagnostics enable the accurate and rapid identification of any pathogen in clinical settings. The CRISPR-Cas system has been used for the development of novel therapeutics against many viruses and it may serve as an advanced-stage therapeutic option for treating HIV infections (Huang et al., 2017; Xu et al., 2019; Herrera-Carrillo et al., 2020).

### Applications of CRISPR-Cas as a Therapeutic Modality Against Infections Caused by ssRNA Viruses

**Figure 2:**
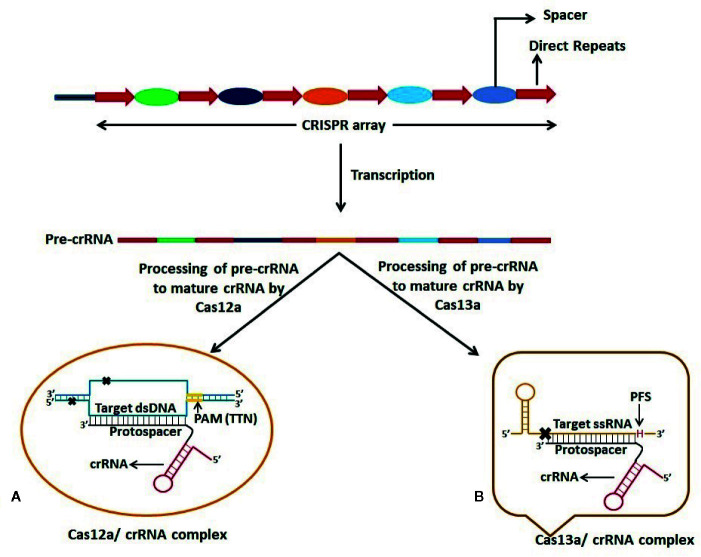
Schematic representation of dsDNA and ssRNA targeting by Cas12a and Cas13a respectively. CRISPR-Cas12a and CRISPR-Cas13a systems possess dual function viz. processing of pre-CRISPR RNA (pre-crRNA) into mature crRNA and cleavage of target nucleic acid. **(A)** Cas12a requires T-rich Protospacer Adjacent Motif (PAM) sequence at 5’ end of the Protospacer which is complementary to one strand of the target dsDNA. Cas12a gives a staggered cut on the dsDNA generating 5’ 4-5 nucleotide overhang distal to the PAM site. **(B)** Cas13a protein recognizes the stem-loop region of crRNA to form Cas13a:crRNA complex and specific cleavage of target ssRNA occurs on the basis of complementarity between the protospacer region and the ssRNA. Cleavage of ssRNA by Cas13a also depends on the presence of 3’ H (A/C/U: Protospacer Flanking Site (PFS)) immediately after the protospacer sequence.

The CRISPR-Cas technology has been utilized by molecular biologists for epigenetic regulation in eukaryotic systems. It has been used as a laboratory tool to edit DNA/RNA for fixing genetic defects and enhancing genetic traits (**Figure 2**). Scientific communities across the world have also adopted this technology to eliminate viruses in human cells. Recently, Freije et al. (2019) at the Broad Institute of the Massachusetts Institute of Technology and Harvard University, USA, demonstrated the potential use of the Cas13 enzyme in cell culture to inhibit the replication of three ssRNA viruses viz. lymphocytic choriomeningitis virus, influenza A virus and vesicular stomatitis virus (Freije et al., 2019). The group identified potential Cas13 crRNA target sites that are highly conserved in the viral RNA and later on used cell culture models to obtain a collection of antiviral crRNAs that could be multiplexed in a combinatorial fashion. The efficient reduction of viral RNA in mammalian cells was shown by the crRNA-directed Cas13 enzyme (Freije et al., 2019). When compared to other nucleic acid-based therapeutics such as shRNAs, a similar viral inhibition was observed by using the CRISPR-Cas13 system.

The group also demonstrated that multiplexing in the form of CRISPR array or multiple crRNAs could be a powerful advantage of using Cas13-based therapeutics. Cas13 could process the CRISPR array to yield individual crRNA for targeting multiple viral RNAs. There have been reports on the induction of mutations in target sites by CRISPR-based antivirals using Cas9 (Wang et al., 2018; Wang et al., 2020), while no crRNA target-site mutation was observed following Cas13 treatment (Freije et al., 2019). These reports indicate the potential of the Cas13 enzyme as an effective antiviral to manage SARS-CoV-2 infection.

### Applications of CRISPR-Cas as a Therapeutic Modality With Regard to COVID-19 and Future Prospects

As of now, any SARS-CoV-2 specific therapy is not available to cure COVID-19 and also, the vaccine development is estimated to take 12–18 months. Recently, CRISPR-Cas13 has emerged as a potent system having the abilityto protect host bacterial cells against bacteriophage infection by using sequence specific crRNAs (Yan and Finnigan, 2018; Yan W. X. et al., 2019). A similar strategy may also be applied to design a therapeutic agent to target the ssRNA genome of SARS-CoV-2 for managing COVID-19.

Recently, Nguyen et al. (2020) implemented the CRISPR/Cas13d system to target RNA molecules, and they have further proposed the use of this system to specifically degrade the SARS-CoV-2 RNA genome by designing crRNAs targeted to the ORF1ab (replicase-transcriptase) and S (spike) genes of the virus (Nguyen et al., 2020). The RdRp gene of SARS-CoV-2 virus can also be an optimal target for CRISPR-Cas13-mediated virus RNA degradation, as the amino acid sequence and molecular structure of RdRp is highly conserved (Buonaguro et al., 2020). The unique feature of the Cas13d enzyme is that its cleavage activity does not depend on PAM-like sequences, and this feature facilitates the targeting of constantly evolving virus variants by rapid development of crRNAs. The group has proposed to deliver the Cas13d effector *via* recombinant adeno-associated virus (AAV) in patients suffering from COVID-19. The small size of the Cas13d effector is expected to facilitate the AAV to carry the CRISPR array containing more than two crRNAs, ensuring efficient virus clearance and reducing the probability of the generation of resistant strains. Moreover, the availability of an AAV serotype having lung tissue specificity can facilitate the targeted delivery of CRISPR.

A CRISPR-Cas13-based strategy for COVID-19 therapy termed Prophylactic Antiviral CRISPR in huMAN cells (PAC-MAN) has been proposed by Abbott et al. (2020) to inhibit SARS-CoV-2. Effective degradation of genomic sequences of SARS-CoV-2 and influenza A virus (IAV) has been demonstrated in human lung epithelial cells (Abbott et al., 2020). The PAC-MAN approach could be a potential antiviral strategy to manage the emerging viral strains and could be implemented rapidly during a pandemic situation. However, *in vivo* delivery of PAC-MAN to the target organ is a big challenge and needs to be addressed to achieve the best result. Viral vectors including AAV, adenovirus and lentiviral vectors may be opted as vehicles for delivery of such CRISPR based antivirals (Lino et al., 2018). Although non-viral vectors are not as prominent as viral vectors, vectors like gold nanoparticles, lipoplexes/polyplexes, cell penetrating peptides, lipid nanoparticles etc. also possess several advantages like ease of scale-up, lack of immunogenicity and delivery of CRISPR-Cas system as RNPs which minimizes the off-target effects (Wilbie et al., 2019). The CRISPR-Cas13 based system targets highly conserved regions in the viral genome thus enabling the system to act against the rapidly mutating strains of the virus.

### CRISPR-Based Applications for COVID-19 Diagnosis

CRISPR-Cas systems have shown their potential to be utilized as molecular diagnostics for detecting nucleic acids (Pardee et al., 2016; Jia et al., 2020) and may replace PCR in many applications. CRISPR-Cas-based diagnostic tools are characterized by sensitivity and specificity comparable to those of traditional PCR, but since they do not require sophisticated, and therefore, expensive equipment, they are associated with meager estimated costs. The inclusion of CRISPR-Cas into molecular diagnostics may reshape the profile of global diagnostic and health care systems (Gootenberg et al., 2017; Chertow, 2018). The Cas proteins to be used in CRISPR-Cas systems vary according to the target nucleic acid (whether the aim is DNA or RNA) and as per the desired applications (Makarova et al., 2015). The CRISPR/Cas system has also been applied to develop CRISPR/Cas9-mediated lateral flow nucleic acid assay (CASLFA) which has been used for the detection of pathogens (Wang et al., 2020).

After the worldwide outbreak of the COVID-19 pandemic, there is an immediate need for quick and easy-to-use diagnostic techniques. CRISPR-based tools may overcome this issue, as these tools have shown significant detection efficiency within 30–60 min; however, they are still awaiting approval from the US Food and Drug Administration (FDA). Recently developed CRISPR-based diagnosis tools using Cas12a or Cas13 nuclease have been following a principle of “collateral cleavage activity”. Cas12a/Cas13 nuclease, a component of the CRISPR tool, gets activated after the CRISPR-RNA (crRNA) targeted cleavage, and once activated, it cleaves all the nearby ssDNA/RNA molecules non-specifically, a feature called collateral cleavage or *trans-*cleavage. Developers have used this property and created fluorescently labeled ssDNA/RNA reporter probes to detect visible bands through the lateral flow assay in a paper strip, making it possible to develop a novel nucleic acid-based diagnostic tool (Chen et al., 2018; Zhang et al., 2020). The crRNA targeted to the viral RNA could activate the Cas protein, leading to the collateral cleavage of the reporter probes and subsequently, the appearance of a positive band on the paper strip (Chen et al., 2018; Zhang et al., 2020).

**Figure 3:**
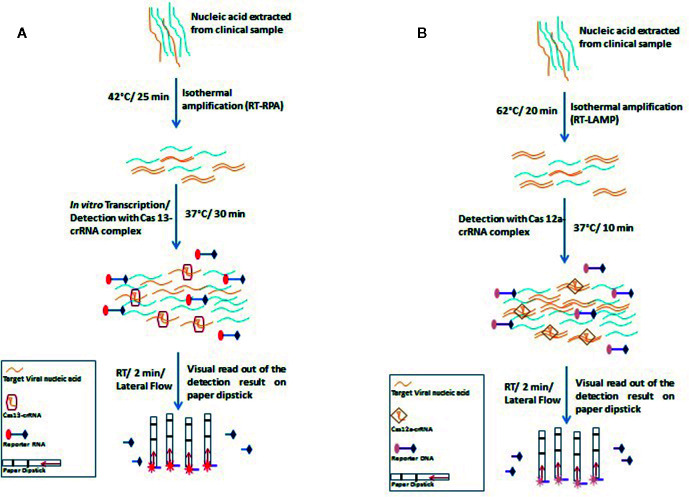
Schematic representation of principles and steps of SHERLOCK **(A)** and DETECTR **(B)**. **(A)** SHERLOCK involves amplification of the pathogenic RNA through reverse transcriptase-recombinase polymerase amplification (RT-RPA) followed by *in vitro* transcription to generate corresponding ssRNA. The ssRNA recognition by Cas13-crRNA complex activates the Cas13 nuclease which further exhibits indiscriminate cleavage of fluorescence tagged reporter ssRNA which is further analyzed on paper strips by lateral flow assay **(B)** DETECTR amplifies the pathogenic RNA through reverse transcriptase- recombinase polymerase amplification (RT-RPA) or reverse transcriptase- loop-mediated isothermal amplification (RT-LAMP). Target (dsDNA) recognition by Cas12a-crRNA complex activates the Cas12a nuclease which further exhibits indiscriminate cleavage of fluorescence tagged reporter ssDNA which is further analyzed on paper strips by lateral flow assay.

The recently invented Specific High-sensitivity Enzymatic Reporter Unlocking (SHERLOCK) technology utilizes the activity of the crRNA-Cas13a protein complex to accurately identify RNA molecules and cleave collateral RNA along with target RNAs. This technology uses non-targeted reporter RNA tagged to a fluorescent dye for the identification of specific RNA molecules (Kellner et al., 2019). The CRISPR-Cas13-based SHERLOCK system has been used to detect SARS-CoV-2 by Zhang et al., who used two crRNAs that recognize two signatures of COVID-19, viz. the S and ORF1ab genes (Zhang et al., 2020). The system searches for virus-specific nucleic acid signatures and provides a visual readout using a test paper strip, similar to a pregnancy kit, within an hour (**Figure 3A**). The SHERLOCK system has also been used by the Sabeti lab, where a website containing CRISPR-Cas13 based assay designs has been created by Metsky et al. to detect 67 viruses which include SARS-CoV-2, Zika virus, and dengue virus, and has the feature to select single or multiplex panels (Chen et al., 2018). This group used synthetic RNA fragments to validate SHERLOCK assay for SARS-CoV-2 detection using both fluorescent and visual readouts, with a sensitivity of 10 copies per microliters. Recently, Sherlock Biosciences has been granted an emergency use authorization (EUA) by the US FDA for its SARS-CoV-2 specific diagnostic assay, which is based on the SHERLOCK technology. This molecular diagnostic assay is expected to be inexpensive and can yield results within an hour.

DNA endonuclease-targeted CRISPR trans-reporter (DETECTR) is a similar technique that amplifies the pathogenic DNA through isothermal recombinase polymerase amplification (RPA), and similar to the SHERLOCK system, reverse transcription is combined in the process for detecting RNA viruses. Target recognition by Cas12a-crRNA complex activates the Cas12a nuclease which further exhibits indiscriminate cleavage of fluorescence tagged reporter ssDNA. DETECTR has successfully been used to differentiate between human papillomavirus 16 (HPV16) and human papillomavirus 18 (HPV18) within an hour in the crude DNA isolated from cultured human cells as well as from clinical samples (Myhrvold et al., 2018).

For COVID-19 diagnosis, Broughton et al. chose two specific crRNAs targeted to the E and N genes, with a detection range of 70–300 copies per microliter of sample input. During the isothermal amplification step, instead of RPA-based amplification, Broughton et al. used loop-mediated isothermal amplification (LAMP) in combination with reverse transcription to diagnose COVID-19 in just 30 min (**Figure 3B**) (Broughton et al., 2020).

All the above mentioned nucleic acid detection methods based on CRISPR-Cas system involve separate nucleic acid amplification and require multiple manual operations thus complicating the detection procedures and increasing the chances of carry over contaminations. Ding et al. developed the All-In-One Dual CRISPR-Cas12a (AIOD-CRISPR) assay method which facilitates highly specific, ultrasensitive and faster visual detection of viral nucleic acids. In this assay, all the reagents required for the detection of viral nucleic acids are incubated at 37°C in one pot, which simplifies the detection procedure and reduces the chances of carryover contamination. The AIOD-CRISPR assay method has been utilized to detect the genomic RNA of HIV and SARS-CoV-2 with high sensitivity within an hour (Ding et al., 2020; Zhang et al., 2020). The assay does not require any lateral flow-based paper dipstick, and instead, the visual detection can be accomplished by imaging the tubes in an LED blue light illuminator. Next-generation point-of-care molecular diagnostic tools for COVID-19 can be developed using this method has the potential for the development of. Researchers in India have identified a Cas9 ortholog from *Francisella novicida* (FnCas9) that is very sensitive to nucleotide mismatches and developed the FnCas9 Editor Linked Uniform Detection Assay (FELUDA) for the diagnosis of SARS-CoV-2 infection in clinical settings as a point-of-care low-cost test (Azhar et al., 2020).

## Limitations of CRISPR-Cas Based Diagnostic and Therapeutic Tools

Management of SARS-CoV-2 infection requires earliest home isolation or social distancing to prevent community spread (check its spread to the community). Therefore, a diagnostic kit having rapid and early detection ability, as well as sensitivity for very low viral titre, is necessarily required. SHERLOCK system is sensitive for around 2 attomolar [2 aM] of DNA and RNA, i.e., around 1cp/µl while the average sensitivity for an existing qRT-PCR method is 6.25 cp/µl (Kellner et al., 2019); LabCorp COVID-19 RT-PCR tests EUA Summary (https://www.fda.gov/media/136151/download) (LabCorp). Moreover, unlike PCR based methods, CRISPR-based nucleic acid detection methods can work at a constant temperature of 37°C. However, SHERLOCK system is limited by exponential pre-amplification of the target which gets saturated soon after the reaction begins and hence, accurate quantification becomes difficult in real-time (Gootenberg et al., 2018). AIOD-CRISPR assay is a rapid method for the detection of viral nucleic acids but its sensitivity is less than the two-step method and also the experiment optimization is a challenging task. Another critical issue is quantification and precise gene expression profiling, which is required for viral copy number estimation. The qRT-PCR is highly sensitive for such experiments.

**Figure 4:**
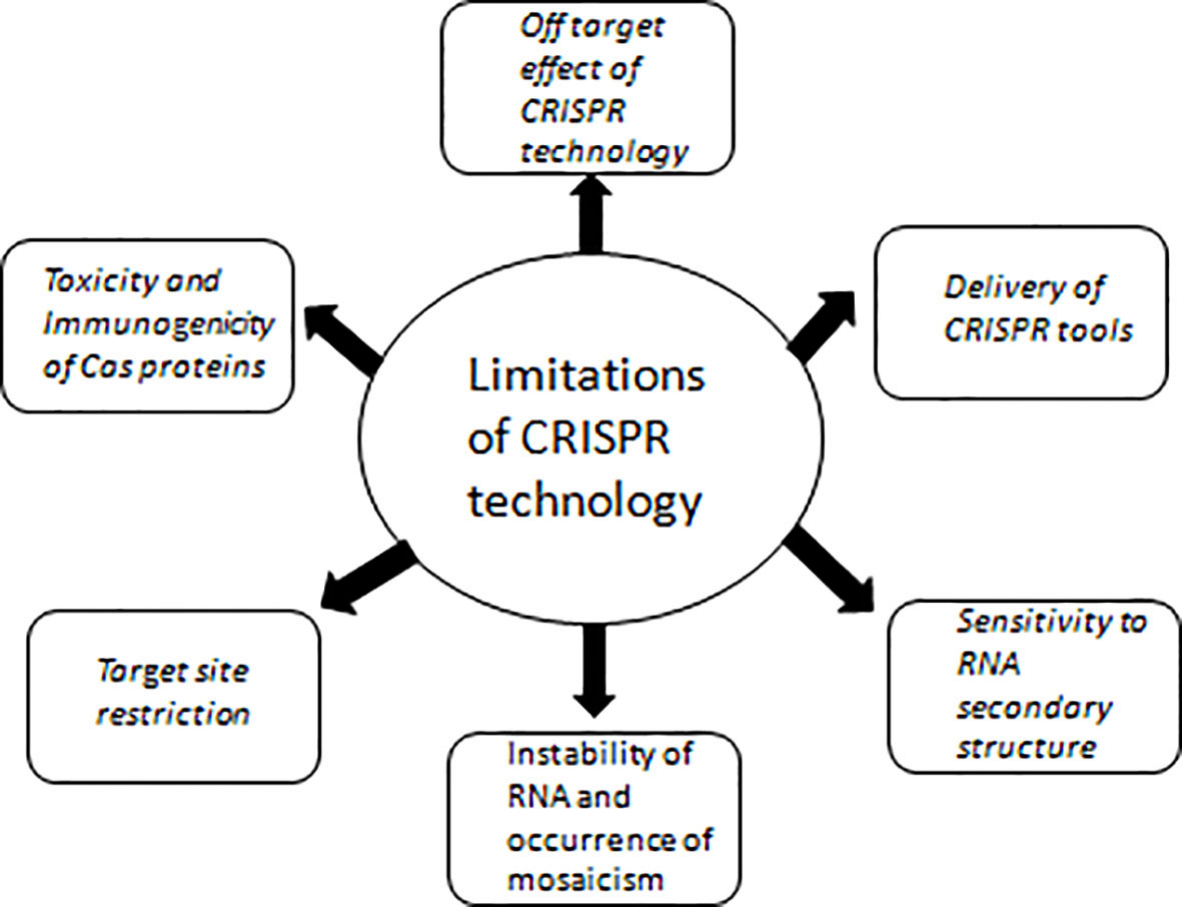
Limitations of CRISPR technology with respect to its therapeutic and diagnostic usage.

As CRISPR-Cas tools are efficient; simple to use, and need low cost infrastructure making it accessible by healthcare workers even in resource limited areas. The advantages of these tools are helping in their adoption in basic and translational research including diagnostic and therapeutic development. Though these tools have several merits, there are drawbacks which are being addressed herein (**Figure 4**). There are many ethical considerations involved in the application of this technology into preclinical or clinical trials. Overcoming the various scientific limitations currently associated with CRISPR/Cas9 mediated editing of the human germline will depend on intensive research that needs to be widely disseminated. In addition, a public discussion on the risks, benefits, and main applications, including all relevant stakeholders, and ultimately guide research priorities in this area must be incorporated.

### Off-Target Effect of CRISPR Technology

The biggest challenge thus far with the CRISPR system is the delivery to the target cells. Non-specific targeting of nucleic acids (with as many as 3 to 5 mismatches) by the CRISPR system has been a matter of concern, especially when it is intended for therapeutic and diagnostic usage (Jin et al., 2019; Zuo et al., 2019). Efficient delivery of the CRISPR/Cas protein tool stands as a major requirement to minimize off target effects within the gene and to ensure that the desired cell or tissue is reached by the tool. Such off-target issues are being addressed by several researchers either by developing off-target detection method or by engineering the CRISPR tools. The off-target detection methods include the use of bioinformatics tools like Cas OFFinder and use of tools like SELEX, DISCOVER, Digenome-Seq, Guide-seq etc, (Kim et al., 2015; Koo et al., 2015; Wienert et al., 2019). Engineering of CRISPR tools include structural modifications in the Cas proteins so as to enhance the specificity for target nucleic acids. Use of variant Cas protein from *Staphylococcus aureus* (SaCas9) having novel mutation (Mut268) could reduce the off-target effects while maintaining the functionality of the protein (Xie et al., 2020). A modified version of Cas9 (Cas9 nickase), which could create nick in only one strand of dsDNA, was also shown to reduce the off-target effects of the system (Satomura et al., 2017). It is uncertain whether modified organisms will be affected indefinitely and whether the correction will be inherited. Further, the qEva-CRISPR method has shown several advantages but it cannot be used for a whole-genome analysis and does not allow for detection of all potential and accidental off-target sites, and does not characterize the identified mutations. In addition, qEva-CRISPR does not allow for reliable detection of a mutation that occurs in a very small fraction (∼5%) of targets.

The safety and efficacy assessment need to be monitored closely during the ongoing studies. A potential risk of using of CRISPR is the introduction of off-target changes to the genome sequence, and thus improving methods for detection of rare mutations and quantifying their potential risks will be important for future clinical advancement

### Delivery of CRISPR Tools

Therapeutic applications of CRISPR technology include the delivery of various tools inside the target cells under *in vivo* condition. Viral vectors are the most preferred vehicle for delivery of these tools and adeno-associated virus (AAV) have been a vector of choice by several researchers but these recombinant viruses show limitations in the size of foreign genes to be carried by them. Most of the Cas proteins have high molecular weight and therefore, it’s a challenge to generate recombinant AAV carrying the gene encoding Cas proteins along with crRNA/gRNA. To overcome this challenge, several low molecular weight Cas proteins viz. Cas14 cjCas9, ScCas9 etc. have been discovered which can also be used for the intended use and can also be delivered by rAAV (Kim et al., 2017; Ibraheim et al., 2018; Karvelis et al., 2019; Li et al., 2019). Though there are merits of viral vectors but *in vivo* delivery using viral vectors suffer from limitations like immunogenicity and duration of Cas expression. However, non-viral vectors which are based on lipids and inorganic particles are devoid of such limitations and could be investigated as alternative for delivery of CRISPR-cas systems for therapeutic purpose (Wilbie et al., 2019).

### Toxicity and Immunogenicity of Cas Proteins

Cas proteins are derived from prokaryotic sources and therefore, *in vivo* delivery of these proteins may result in toxicity in the human cells harboring these proteins and also in immune activation leading to production of Cas protein specific antibodies (Charlesworth et al., 2019). These antibodies may interfere in the therapeutic use of CRISPR technology. Moreover, various Cas proteins are derived from human pathogens and prior activation of immune system by these pathogens might hinder the use of such CRISPR tools. It has been shown that incorporation of two mutations in epitope anchor residues of cas9 protein reduces its immunogenicity (Ferdosi et al., 2019). Further, it is observed that the terminal phosphate group in gRNA/crRNA can lead to innate immune response (Kim et al., 2019) and removal/modification of this terminal group of these nucleic acids might increase the efficiency of CRISPR technology.

### Target Site Restriction

Several Cas proteins including Cas9 and Cas12a need PAM sequence for targeting a specific region in the target genome. The PAM sequences are highly specific for the type of CRISPR-Cas system and unavailability of specific PAM sequence near the target region greatly hinders the use of this technology for therapeutic purpose (Moon et al., 2019). Flexibility in the target selection by these CRISPR tools is required to enhance their efficiency. Towards this end, study involving generation of PAM variants of Cas9 and Cas12a have been done which could recognize more than one PAM (Kleinstiver et al., 2015; Gao et al., 2017). Engineering other important Cas proteins like Cas13 variants is also required to increase their efficiency and specificity.

### Sensitivity to RNA Secondary Structure

RNA generally acquires secondary structure inside the cells and this can affect the efficiency of RNA specific Cas proteins like Cas13. Elucidation of mechanisms behind the sensitivity of Cas13 proteins could help in engineering these proteins so as to make them less sensitive to RNA secondary structure (Wolter and Puchta, 2018).

### RNA Instability and Occurrence of Mosaicism

Fragile nature of RNA due to ubiquitous presence of RNase may significantly affect the efficiency of the CRISPR based diagnostic system. Other limitations in the use of CRISPR system for therapeutic applications include occurrence of mosaicism which happens when transduced cells divide before the editing or cleavage of target nucleic acids (Yen et al., 2014).

## Conclusion and Future Prospects

In the modern era of science and technology, although significant advancements have been made in the field of infectious diseases, human beings become helpless whenever novel virulent viruses such as SARS-CoV-2 emerge in an unsuspecting population. Now, as per the current prevailing situations where the major hurdles are the requirements of specific equipment, the time-consuming processes associated with disease diagnosis and limited availability of diagnostic centers, there is an urgent need for such low-cost, portable, rapid, and easy-to-use diagnostic techniques that could be applicable in resource limited remote areas which are at higher risk of SARS-CoV-2 transmission, including airports and local community hospitals in low- to middle-income countries. Since SARS-CoV-2 infection causes flu-like symptoms, the accurate diagnosis of such infections is essential to control the dissemination of the virus within a population and beyond the affected people. Moreover, SARS-CoV-2 causes symptomatic as well as asymptomatic infection, and hence, it has been able to cross the territories without getting noticed in the infected but asymptomatic individuals. Such individuals are not screened, and they keep shedding the virus in the environment, infecting several other individuals around them. The availability of an affordable and portable diagnostic system may aid in extensive scale screening of affected populations to stop the chain of transmission of highly infectious viral infection.

CRISPR-Cas system is a next-generation technology helping in to develop novel highly specific anti-viral therapeutics and molecular diagnostics with rapid, accurate, point-of-care use without any need for technical expertise and expensive equipment in resource poor setting. CRISPR based diagnosis could fulfil this requirement being easy to use, portable, and take less time than existing real-time PCR based standard diagnosis method for the diagnosis of COVID-19. Though CRISPR based therapies are still not approved, the CRISPR based diagnostic has obtained the US FDA emergency use authorization licence. Currently, the screening and diagnosis of COVID-19 are being done by real time RT-PCR, which detects viral RNA in respiratory samples. Though the diagnosis is specific and sensitive, but infrastructure requirements, need of trained manpower, and cost are significant limitations. CRISPR-Cas technology proved highly effective in developing diagnostics against some viruses like dengue, Zika, etc. Diagnostics based on CRISPR-Cas technology like DETECTR and AIOD-CRISPR has been shown to be fast, reliable, and cost-effective. Although not yet commercialized, the paper-based diagnostics can be a very easy to use tool to diagnose the virus infection at the point-of-care and in resource-limited remote areas. Large scale screening of affected populations can be done using such diagnostic tools, which will help in routine surveillance and restricting the community spread of this virus. The development of therapeutic drugs against RNA viruses is a great challenge as favourable mutations keep on accumulating in their genome, leading to the development of antiviral resistance. To face the challenges, strategies have been explored for the development of therapeutics targeting CRISPR-Cas technology, which targets not only the viral genome but also the host machinery involved in virus replication. As CRISPR mediated abrogation of CCR5 co-receptor gene conferred resistance to CD4 T cells for HIV infection, similar approach can be adopted to make the host cell resistant to SARS-CoV-2 by delivering the CRISPR-Cas13d into the susceptible host cells. The proposed strategy of CRISPR based therapeutics for COVID-19 are next generation technologies with significant challenges in terms of delivery, safety, efficacy and regulatory approval for use in human subjects. Delivery remains the biggest obstacle in the use of CRISPR based therapeutics. Several methods have been explored for the delivery of CRISPR with each method having some merits as well as demerits. Among the various approaches available for targeting nucleic acids, delivery of ribonucleoproteins, rather than plasmid DNA or mRNA, is associated with minimal off-target effects (Zuris et al., 2015). Therefore, development of approaches for effective and targeted delivery of RNPs may have a significant impact on CRISPR based therapeutics development. We further need to assess the fate of components of delivery system in the body, their duration of stay in body and toxicity of each component. The assessment of potential risks associated with use of CRISPR will be important for future clinical advancements.

## Author Contributions 

All authors contributed to the article and approved the submitted version.

## Conflict of Interest

The authors declare that the research was conducted in the absence of any commercial or financial relationships that could be construed as a potential conflict of interest.
